# Identification of mosquito repellent odours from *Ocimum forskolei*

**DOI:** 10.1186/1756-3305-4-183

**Published:** 2011-09-22

**Authors:** Teun Dekker, Rickard Ignell, Maedot Ghebru, Robert Glinwood, Richard Hopkins

**Affiliations:** 1 Division of Chemical Ecology, Department of Crop Science, Swedish University of Agricultural Sciences PO 44, Alnarp, SE-23053 Sweden; 2 Department of Biology, University of Asmara, Asmara, Eritrea; 3 Department of Ecology, Swedish University of Agricultural Sciences, Box 7044, Uppsala SE-750 07, Sweden

**Keywords:** *Ocimum forskolei*, Lamiaceae, GC-EAD, *Aedes aegypti*, mosquito, repellent, linalool, methyl cinnamate, methyl salicylate

## Abstract

**Background:**

Native mosquito repellent plants have a good potential for integrated mosquito control in local settings. *Ocimum forskolei*, Lamiaceae, is used in Eritrea as a spatial mosquito repellent inside houses, either through crushing fresh plants or burning dry plants. We verified whether active repellent compounds could be identified using gas-chromatography coupled electroantennogram recordings (GC-EAD) with headspace extracts of crushed plants.

**Results:**

EAD active compounds included (R)-(-)-linalool, (S)-(+)-1-octen-3-ol, trans-caryophyllene, naphthalene, methyl salicylate, (R)-(-)-α-copaene, methyl cinnamate and (E)-ocimene. Of these compounds (R)-(-)-linalool, methyl cinnamate and methyl salicylate reduced landing of female *Aedes aegypti *on human skin-odor baited tubes. The latter two are novel mosquito repellent compounds.

**Conclusions:**

The identification of mosquito repellent compounds contributes to deciphering the mechanisms underlying repulsion, supporting the rational design of novel repellents. The three mosquito repellent compounds identified in this study are structurally dissimilar, which may indicate involvement of different sensory neurons in repulsion. Repulsion may well be enhanced through combining different repellent plants (or their synthetic mimics), and can be a locally sustainable part in mosquito control efforts.

## Background

For blood feeding insects, olfaction is the principal sensory modality used in host recognition and location. This is especially true for disease vector mosquitoes, many species of which locate their vertebrate hosts during scotophase [1]. Despite much research, mosquitoes remain a huge burden on society, both as nuisance pests and as vectors of disease. Tropical regions are particularly affected, largely due to the economic and logistical problems associated with conventional control methods, such as insecticide sprays. Although early efforts to control vectors with insecticides were highly effective, their reliance on spraying inside houses to kill resting females has raised environmental and public health concerns [2]. In addition, the contrasting behaviour of different mosquito vector species leads to variation in the effectiveness of different methods in controlling local mosquito populations [e.g., [3]]. Hence, there is a need for integrated sets of control methods adapted to local settings, which can be provided at minimal cost and are thus accessible to local people. One such key method may be the use of locally available plants, traditionally used to deter mosquitoes [4].

Synthetic repellents, such as DEET (N, N, diethyl-toulamide), are commonly used for personal protection from nuisance biting and may also reduce disease transmission if they reduce biting sufficiently [5]. However, synthetic repellents are often too expensive for people living in rural areas in the tropics. As an alternative, the use of plants as insect repellents dates back more than 2000 years [6], and a wide range of plants have been used to repel mosquitoes [7]. These plants may be effective when burnt to produce smoke [e.g., [8,9]], or placed as potted plants inside houses [e.g., [10]]. *Ocimum *spp. have been documented as traditional repellent plants, and are effective against mosquitoes [9,11-15], black flies [16] and ticks [17]. *Ocimum forskolei *is used as a mosquito repellent in the Western lowlands of Eritrea where it grows widely in bush land at an altitude of 100-1000 m. In field studies, *O*. *forskolei *hung at the head and foot of the bed in houses, reduced *Anopheles arabiensis *mosquitoes bites by 53% [14]. Furthermore, laboratory wind tunnel studies found that when crushed *O. forskolei *was added to human odour, it became less attractive than human or goat odour alone [18].

Improved knowledge of the volatile compounds released by *O*. *forskolei *may improve the use of the plant in field situations. It may also lead to the identification of novel compounds with potential for development as synthetic repellents, as well as deciphering the olfactory mechanisms underlying repulsion. The specific questions addressed in this study are: 1) what are the principal components of the volatile odour of crushed *O*. *forskolei *that are detected by mosquitoes, and 2) are any of these compounds released by *O. forskolei *involved in repelling *Aedes aegypti*?

## Methods

### Colony

*Aedes aegypti *Rockefeller strain were used in all experiments (eggs courtesy of Dr. W. Takken, Wageningen University, The Netherlands). Mosquitoes were reared at 25°C, 80% RH and a L:D cycle of 12:12 hours without an artificial dusk period. Larvae were reared on Tetramin^® ^fish food. Pupae were collected into bowls and placed in cages for adults to emerge; males and females were allowed to mate. Adults were kept in cylindrical cages of 20 cm (diameter) by 30 cm (height), and provided with a 6% glucose solution. Females were offered a human arm for blood feeding.

### Collection of volatiles from *O. forskolei*

Plant material was entrained inside glass bell jars (4 l capacity), resting on circular aluminium plates (210 mm diameter × 3 mm thick). The neck opening was sealed with a stopper made from inert silicone rubber containing three holes of 6 mm diameter for the absorbent tube, air inlet line, and one open hole. Prior to volatile collection, the bell jars were washed with detergent, rinsed with acetone and distilled water and baked overnight (15 hours) at 200°C. The aluminium plates were washed with detergent and baked overnight (15 hours) at 200°C. Collected volatiles were absorbed on Porapak Q (60 mg, 80-100 mesh) contained in glass GC inlet liners (80 mm × 3 mm ID, ATAS GL, Netherlands), held in place with glass wool plugs. Prior to volatile collection, Porapak tubes were washed with freshly redistilled diethyl ether and conditioned by heating overnight (15 hours) at 200°C under a stream of nitrogen. Air for the entrainment was purified by passing it through molecular sieve (10 Å) and activated charcoal traps (10-18 mesh) before entering the bell jars. Filters were baked overnight at 200°C under a stream of nitrogen.

Collections were performed in a controlled environment chamber, at 21 ± 1°C, 60% RH, and constant lighting. *O. forskolei *(18 g stem and leaves removed from four-month old plants) was crushed by hand in the typical manner used in Eritrean homes, and was placed inside the bell jar at around 9 am (3 hours into the photoperiod). Air was pumped into the jar, through the filters, at a rate of 1200 ml/min for 1 hour to disperse contaminated air, after which the absorbent tube was introduced and air drawn through the absorbent at a rate of 700 ml/min. The vessel was therefore under positive pressure, and the remaining 500 ml/min air was vented through the extra hole in the silicone stopper. Collections were made for 2 hours. Collected volatiles were eluted from the Porapak Q with 750 μl freshly redistilled diethyl ether and stored at -20°C. Prior to GC-EAD and GC-MS, samples were concentrated under a gentle stream of nitrogen to 10% of their original volume.

### GC, GC-EAD, GC-MS

A Hewlett Packard HP6890 gas chromatograph (GC, Santa Clara, Ca) was fitted with a 30 m × 0.25 mm ID Solgelwax column (SGE, 30 m × 0.25 mm i.d., 0.25 μm stationary phase, Agilent Santa Clara, Ca), which has superior separation capabilities for polar compounds. Nitrogen was used as carrier gas for the GC, and helium for the GC-mass spectrometer (MS). One μl of the headspace volatile collection was injected onto the column. A fast program was used: 40°C (initial time) for 2 min., ramp: 10°C/min, final time 220°C for 5 min. The GC outlet was split into two. One portion was sent to a flame ionization detector (FID), whereas another portion was led into the air stream over the mosquito head. GC-traces and antennal responses were visualized on a computer monitor. Co-occurring FID peaks and antennal responses were identified using a HP 5970 GC-MS fitted with a solgelwax column (Agilent). Chirality of the compounds was verified by injecting the sample and standards in a HP-chiral column.

### Electro-antennogram recordings

For electro-antennogram (EAG) recordings, we used unfed, mated, 5-10 day-old female mosquitoes. A pipette tip with half a mosquito's head including the antennae protruding was vertically mounted under an Olympus microscope at 400 × magnification. Glass capillaries with a silver wire were filled with Beadle-Ephrussi ringer solution [19]. The recording glass electrode was placed at the tip of the flagellum, and the reference electrode was inserted through the eye. The EAG signal was pre-amplified 10 × using a Syntech probe (Syntech, Kirchzarten, Germany). A Syntech I/O box combined signals from different channels (EAG, stimulus, GC-trigger and GC). After A/D conversion (Syntech IDAC PCI card), the signals were visualized and saved on a PC with Syntech software.

### Landing assays

Thirty to thirty-five *Ae. aegypti *females (5-10 days old) were released into the landing assay cage (26 ± 1°C and 65 ± 5% RH) 3 hours prior to the experiments, without access to sugar solution. Landing assays were performed in a 30 cm cubic gauze cage (BugDorm, MegaView, Taiwan), with one side replaced by a Plexiglas panel with two holes through which landing tubes could be inserted. Landing tubes were standard test tubes, diameter 25 mm, length 150 mm, which were sandblasted to improve odour adhesion. The tubes were heated by two metal rods at 40°C inserted into the glass tubes at the start of the experiment. Two landing tubes were rubbed through the experimenter's palms for 2 minutes. The tubes were exchanged between the two hands every 15 seconds. Ninety μl of a test odour (see below) was spread onto one tube using a micropipette, and hexane was similarly applied on the control tube. The tubes were inserted through the holes in the cage and the number of mosquitoes landing on the tubes was scored every 30 seconds during the ensuing 90 seconds. In between each experiment with a test odour, a control test was carried out with two tubes with only human odour rubbings. The tube holders were wiped with ethanol after each experiment. The time between two consecutive trials was 15 minutes. Mosquitoes were used for a maximum of eight consecutive trials. The sequence of odours offered on the tubes was randomized. Generally between 60-80% of the mosquitoes landed on the tubes, but if fewer than 50% of the mosquitoes responded (measured between each trial as a control test), the test was terminated and a new batch of mosquitoes was used.

The following odours were tested (purity, CAS number and company from which they were purchased): linalool (+/-, > 97%, CAS 78-70-6, Aldrich); 1-octen-3-ol (> 98% purity, CAS 3391-86-4, Aldrich); trans-caryophyllene (> 98.5, CAS 87-44-5, Aldrich); naphthalene (> 99%, CAS 91-20-3, Fluka); methyl salicylate (> 98%, CAS 119-36-8, Aldrich); (-)-α-copaene (> 96%, CAS 3856-25-5, Sigma); methyl cinnamate (> 99%, CAS 103-26-4, Fluka); ocimene (E:Z:allo-, 28:62:3, CAS 13877-91-3, International Flavors and Fragrances). Odours were diluted in decadic steps in hexane.

### Statistical analysis

Counts of landings over time in treatment tests and control test were summed over 90 seconds and fit to a logit function using treatment and test number as factors. The significance of factors in the contribution to the model was statistically tested using a X^2 ^distribution.

## Results and Discussion

### Headspace composition of O. forskolei

**Figure 1:**
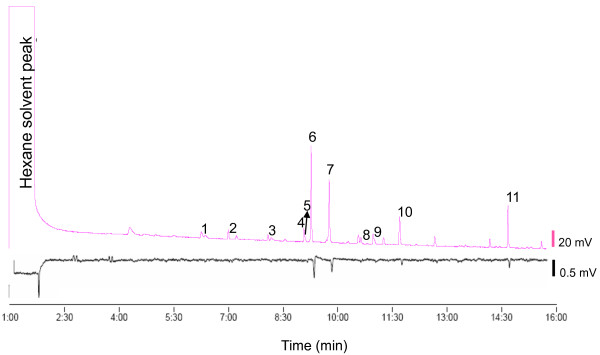
**Sample GC-EAD trace**. The upper trace illustrates GC peaks for (1) (E)-ocimene, (2) unknown, and 4-hexen-1-ol- acetate, (3) 3-hexenol, (4) 1-octen-3-ol, (5) α-copaene, (6) linalool, (7) trans-caryophyllene, (8) unknown sesquiterpene, and α-caryophyllene, (9) naphthalene, (10) methyl salicylate, (11) methyl cinnamate. The lower trace show the antennal response to eluding compounds. Not all individuals were equally responsive.

In this study we tested whether GC-EAD analyses can be used to identify repellent compounds from headspace extracts of repellent plants. We found that a number of compounds in *O. forskolei *extracts elicited consistent electrophysiological responses in antennae of *Ae. aegypti *(Figure 1). Eight candidate compounds were identified through GC-MS analyses and selected for further landing bioassays, including (S)-(-)-linalool, naphthalene, methyl salicylate, (S)-(+)-1-octen-3-ol, (R)-(-)-α-copaene, (E)-ocimene, methyl cinnamate and trans-caryophyllene.

The compounds that we identified in this study are found in a number of other *Ocimum *species in varying ratios. Kéita et al. [20] investigated three *Ocimum *spp., and found that the essential oil of *O. basilicum *was dominated by around 70% linalool, whereas *O. gratissimum *and *O. suave *had little or no linalool, but the former around 1% alpha-caryophyllene and the latter around 1% beta-caryophyllene. Jirovetz et al. [21] also found *O basilicum *essential oil to be characterised by linalool (30%), though less than 1% was found in *O. americanum*, *O. gratissimum *and *O. sanctum*. Methyl cinnamate was dominant in *O. americanum *and *O. basilicum*, but not detected in *O. gratissimum *and *O. sanctum*, beta-caryophyllene made up 16% of the essential oil of the latter. Kasali et al. [22] found that *O. basilicum *contained 10.8% linalool and 6.3% (Z)-methyl cinnamate. Analysis of the essential oils from twelve varieties of basil (*Ocimum *spp.) found that ten of them were characterized by a high percentage of methyl cinnamate, ranging from 35% to 80% [23]. Of the other two, one was characterised by a high concentration of linalool and the other by a high concentration of caryophyllene.

### O. forskolei volatiles suppress landing of **Ae. aegypti**

**Figure 2:**
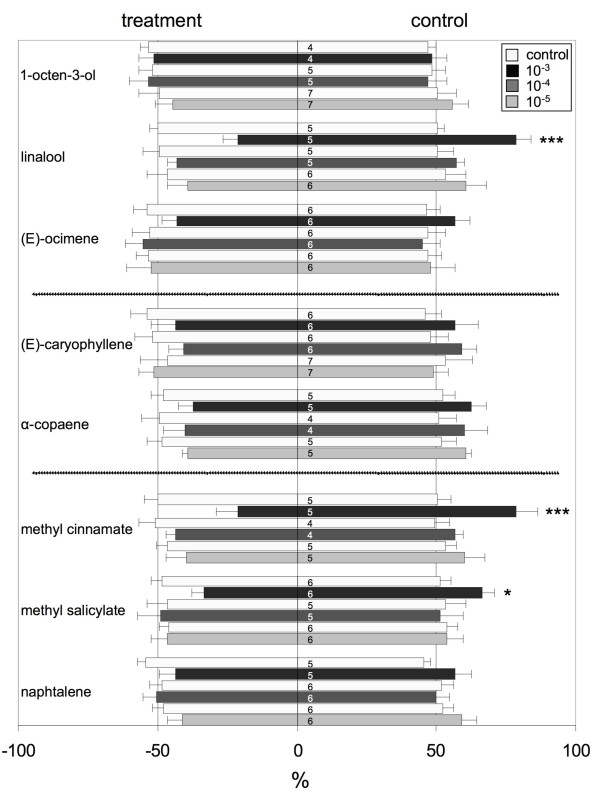
**Percentage ± SE of *Aedes aegypti *females landing on the treatment (left) and control (right) tubes during a 90 s. experimental period**. Each bar represents 100%. A treatment was preceded by a control test to check for mosquito responsiveness and for symmetry in preference (white bars). *P < 0.05, ***P < 0.001.

We subsequently tested which of the volatile compounds identified in the headspace collection of *O. forskolei *reduced landing in bioassays with *Ae. aegypti*. Most compounds did not show evidence of mosquito repellency at any of the concentrations tested (10^-3 ^to 10^-5^). These include 1-octen-3-ol, (E)-ocimene, naphthalene, caryopyllene and α-copaene. In contrast, the oxygenated monoterpene linalool, and the benzenoids methyl cinnamate and methyl salicylate did reduce landing at a concentration of 10^-3 ^(Χ^2 ^DF = 5, P < 0.001, P < 0.001, and P = 0.012, resp. Figure 2). No significant effects of test number on repulsion were recorded. The model using treatment and day as factors sufficiently explained the variance in all comparisons (Pearson's Goodness-of-Fit, P > 0.1 in all cases). Lower concentrations of these compounds did not reduce landing rates compared to the control. Control experiments interspersed by actual tests (white bars in Figure 2) demonstrate that the choice of mosquitoes between the two baited glass rods was symmetrical, i.e., there was no bias for either side of the cage (Figure 2, white bars).

Our results indicate that the mosquito repellent activity of *O. forskolei *is based on at least three compounds. The compounds, linalool, methyl salicylate and methyl cinnamate all repelled *Ae. aegypti *by 40-70% at 10^-3 ^in a simple landing bioassay. Linalool and methyl cinnamate are commonly reported components of essential oils, and *Ocimum *spp. have been reported to contain varying quantities of these compounds [e.g. [21-23]]. Linalool [24] and caryophyllene [13] are recognised as mosquito repellents, although the latter did not repel mosquitoes in our landing assays. However, methyl cinnamate and methyl salicylate have not previously been described as mosquito repellents. The potential of *O. forskolei *for use against a range of mosquito species is increased by the presence of a number of structurally unrelated active compounds. In the present study, activity was found against *Ae. aegypti*. However, *O. forskolei *was also repellent for *Anopheles arabiensis *in the field [14] and *An. stephensi *under laboratory conditions [18], indicating similar mechanisms of detection, processing and mode of repulsion by these compounds across mosquito genera. The results also demonstrate that electroantennographic detection in combination with landing assays can be used to identify potential repellents, and may indicate a mode of action through activation of olfactory receptor neurons. The technique may thus be used in the identification of repellent compounds from other plants with mosquito repellent properties.

Inasmuch as the compounds identified here are found in most *Ocimum *spp., so are the repellent properties generic across this genus. *O*. *forskolei *reduced biting by over 50% against *An. arabiensis *under field conditions [14], and reduced capture of *An*. *stephensi *in a dual choice olfactometer under laboratory conditions [18]. Fresh *O. canum *is used as a mosquito repellent in native villages in Guinea Bissau in West Africa, and in field tests had a repellency of over 60% [25]. Fresh plant material of *O. americanum *has been used as a mosquito repellent in Kenya [9,10], whereas *O. suave *and *O. kilimandscharium *are used extensively in Tanzania, and are highly effective in bioassays against a range of mosquito species [15]. In addition, there are reports of *Ocimum *spp. being used as mosquito repellents via burning or thermal expulsion [e.g. [10,11,26]], or through the use of essential oils [e.g. [12,13,26]]. Given such a wide range of source material, *Ocimum *spp. merit a systematic investigation of their relative properties. *Ocimum *species differ considerably in their volatile constituents, which could imply that a combination of various species or their synthetic mimics may further augment the repellency of *Ocimum *species.

### Use of mosquito repellent plants and their compounds in mosquito control

Whereas synthetic repellents such as DEET can reduce biting to an extent that can be sufficient to suppress outbreaks of malaria in South Africa [5], there are issues of cost, logistics and toxicity [e.g. [6]], and in some cases of resistance [27,28]. In contrast, native plants have the advantages of low cost and local availability. In addition, *Ocimum *spp. exert their effect at a distance rather than at close range or upon contact, which minimizes the risk of toxicity. However, low toxicity of topical *Ocimum selloi *oil [13] indicates that *Ocimum *spp. may also be suitable as topical repellents.

The use of native plants may be a locally sustainable piece in the puzzle of establishing integrated mosquito vector control. Whilst insecticide treated bed nets provide excellent protection during sleep, repellents protect prior to bedtime. Repellents may especially be effective against opportunistic or zoophilic mosquitoes, which can be diverted to an alternative host. The repellent activity reported here for *O. forskolei *is clearly only a small part of the potential available within *Ocimum *spp. Selection and combination of different strains or *Ocimum *species may further improve their potential. Their low cost means that they can benefit even the poorest members of a community. This alone makes improving the understanding of such systems a worthy goal.

## Conclusions

This study demonstrates that *O. forskolei *is repellent for a range of mosquito species. Active compounds identified through GC-EAD and GC-MS include R-(-)-linalool, methyl salicylate and methyl cinnamate. The results indicate that repellents can be identified through measuring summed olfactory responses (EAG), which may indicate that these repellents function through activating olfactory receptor neurons. Methyl salicylate and methyl cinnamate are newly described mosquito repellents. Identification of a diverse set of repellent compounds helps in the identification of their mode(s) of action and opens up possibilities for creating superior repellents through combination.

## Competing interests

The authors declare that they have no competing interests.

## Authors' contributions

All authors were involved in the design of the experiments. TD, MW and RG carried out the experiments and analysed the data. TD and RH wrote the first draft of the manuscript. All authors approved the final version of the manuscript.
